# Older adults as active research partners: protocol for an umbrella review

**DOI:** 10.1136/bmjopen-2025-114885

**Published:** 2026-03-18

**Authors:** Ann-Therese Hedqvist, Susanna Strandberg, Bodil Holmberg, Joakim Niklasson, Paola Violasdotter Nilsson, Willemo Carlsson, Maria Andreassen, Sara Bergstrand, Mats Holmberg, Charlotta Nilsen

**Affiliations:** 1 Department of Health and Caring Sciences, Linnaeus University, Kalmar, Sweden; 2 Ambulance Services, Region Kalmar län, Västervik, Sweden; 3 Department of Health and Caring Sciences, Linnaeus University, Växjö, Sweden; 4 Department of Nursing Science, Sophiahemmet University, Stockholm, Sweden; 5 Department of Health, Blekinge Institute of Technology, Karlskrona, Sweden; 6 University Library, Jönköping University, Jönköping, Sweden; 7 Independent Public Contributor, Växjö, Sweden; 8 Department of Health, Medicine and Caring Sciences Rehabilitation and Community Medicine, Linköping University, Linköping, Sweden; 9 Department of Health, Medicine and Caring Sciences, Linköping University, Linköping, Sweden; 10 Department of Ambulance Service, Region Sörmland, Eskilstuna, Sweden; 11 Institute of Gerontology, Jönköping University, Jönköping, Sweden

**Keywords:** Aged, Community-Based Participatory Research, Review, Research Design

## Abstract

**Abstract:**

**Introduction:**

The involvement of older adults as active partners in research is increasingly being promoted to improve the relevance and impact of scientific knowledge. However, the evidence base on how older adults have been involved as active partners in healthcare research remains fragmented. To our knowledge, no review of reviews has yet provided a comprehensive overview of this body of evidence. Therefore, this umbrella review aims to synthesise review-level evidence on the involvement of older adults as active research partners. We address three questions: (1) How have older adults been involved as active partners in research? (2) What terminology, models and frameworks have been used? (3) What benefits and challenges have been reported related to involving older adults as active partners in research?

**Methods and analysis:**

This study will follow the Joanna Briggs Institute (JBI) methodology for umbrella reviews. A comprehensive search will be conducted in Medline, CINAHL, Scopus, PsycINFO, Sociological Abstracts and Web of Science. Eligible reviews will be those reporting on the involvement of older adults (aged 60 years or older) as active partners in research. Two reviewers will independently screen titles, abstracts and full texts and perform data extraction using a standardised form. Methodological quality will be assessed using the JBI Critical Appraisal Checklist for Systematic Reviews. Findings will be synthesised narratively and thematically, with attention to reported roles, terminology, conceptual frameworks and the benefits and challenges of involvement.

**Ethics and dissemination:**

As this umbrella review draws exclusively on secondary data from published sources, ethical approval is not required. Older adults, engaged as independent public contributors, have been involved in shaping the review protocol and will take part in interpreting the findings. Results will be disseminated through a peer-reviewed journal and presentations at academic and stakeholder conferences, and used to inform the design of a subsequent mixed-methods study focused on strengthening the involvement of older adults as active partners in research.

**PROSPERO registration number:**

CRD420251064947.

**STRENGTHS AND LIMITATIONS OF THIS STUDY**

- The review follows rigorous methodology, including the Joanna Briggs Institute guidelines for umbrella reviews and the Preferred Reporting Items for Systematic Reviews and Meta-Analyses reporting frameworks, ensuring methodological transparency and replicability.
- Public contributors (older adults) have been involved throughout the development of the protocol, enhancing its relevance and alignment with lived experience.
- Due to heterogeneity in review designs, findings will likely not be directly comparable, and the synthesis might be confined to a narrative output.
- More recent findings may not be captured, as they may not yet have been included in published reviews.

## Introduction

 Older adults have traditionally been positioned as passive subjects in research, with limited influence over study design, priorities or interpretation of findings.^1^,^5^ However, in recent years, there has been a shift towards recognising members of the public, including older adults, as active partners in research and co-creators of knowledge.^6^,^8^ This development reflects broader efforts to democratise research and enhance its relevance, particularly in the context of ageing, healthcare and social care.

Despite growing recognition of participatory research approaches, several barriers may limit older adults’ meaningful involvement as active research partners. These include physical and cognitive health challenges, digital exclusion, transportation and time constraints, and institutional structures that privilege academic expertise over experiential knowledge.^9^,^12^ Structural and cultural factors, including ageist assumptions and entrenched power imbalances between researchers and public contributors, may further constrain equitable collaboration.^13^ These barriers underscore the need to synthesise existing evidence on how older adults are engaged, and under what conditions their involvement becomes meaningful rather than tokenistic.^14 15^

In this review, the lower age limit defining ‘older adults’ is set at 60 years, in line with the WHO’s classification, which commonly uses 60+ years as the threshold for older age in global health and ageing research.^16 17^ This definition ensures international comparability. We acknowledge that chronological age is an imperfect proxy for ageing. The experience of being older is highly individual, as ageing is shaped by genetics, lifestyle, health status and social factors.^16^,^18^ Therefore, the review adopts an inclusive approach, recognising both the heterogeneity of older populations and the diversity of ageing experiences across contexts.

## Background

Participatory approaches—such as co-creation, co-production, citizen science, community-based participatory research and patient and public involvement (PPI)—aim to engage members of the public, including older adults, as active partners in shaping research agendas, methodologies and outcomes.^15 13 19^,^28^ These approaches highlight the value of lived experience and promote more inclusive, ethical and context-sensitive knowledge production.

Research shows that engaging older adults as active partners in research can improve the quality and relevance of findings, inform more inclusive service design, and support policy development grounded in real-world needs.^29 30^ Involvement may strengthen community engagement and challenge ageist stereotypes.^1020 31^,^34^ However, despite this growing interest, the field remains fragmented, with diverse terminology, inconsistent reporting and varying degrees of involvement.

Engaging older adults as active partners in research may not only improve research outcomes but also promote personal empowerment, skill development and social inclusion.^9 33 35^ For example, Traversa *et al*^36^ demonstrated how involving older adults in co-design processes contributed to more acceptable and sustainable interventions. Despite this increasing momentum, several challenges remain. Studies point to enduring power imbalances between academic researchers and older participants and warn against tokenistic practices where involvement lacks meaningful influence.^14 15^ Key dimensions such as reflexivity, inclusive recruitment strategies and sustained engagement over time are often underdeveloped.^37 38^

Although a substantial body of research has explored PPI more broadly across healthcare and social care,^13 26 39 40^ few studies have focused specifically on older adults. In their early review, Fudge *et al*^4^ highlighted the potential of older adults’ contributions to research in the UK and called for greater inclusion. More recently, studies in Sweden^41 42^ identified broad support for involvement among older adults but also revealed uncertainty about how, when and under what conditions they could participate meaningfully. Overviews have mapped models and impacts of PPI across disciplines,^43^ revealing inconsistent terminology, limited details on how participation is enacted, and a lack of theoretical frameworks to explain how involvement affects research outcomes.

Despite an expanding evidence base, no synthesis has yet captured the breadth of older adults’ involvement as active partners in research across existing literature. Existing syntheses do not address the specific needs, barriers and contributions of older adults. This omission is significant, given that older adults may encounter unique challenges—including ageism, health-related limitations and digital exclusion—while also offering critical perspectives drawn from lived experience of ageing, illness and caregiving.^44 45^ Terminological inconsistency further complicates the field. Terms like co-research, co-design, co-production and citizen science are often used interchangeably, leading to conceptual ambiguity and limiting comparability across studies.^46 47^

Various systematic reviews have explored public involvement in research, but to our knowledge, no review of reviews has yet provided a comprehensive overview of this body of evidence. Therefore, this umbrella review aims to synthesise review-level evidence on the involvement of older adults as active research partners.

### Review questions

The following questions will be addressed:

1. How have older adults been involved as active partners in research?
2. What terminology, models and frameworks have been used to describe the involvement of older adults as active partners in research?
3. What benefits or challenges have been reported related to involving older adults as active partners in research?

## Methods and analysis

### Study design

This study is an umbrella review, defined as a review of existing systematic reviews and meta-analyses that address a shared research question or phenomenon of interest.^48 49^ Umbrella reviews are particularly well-suited to synthesising a broad evidence base and providing a high-level overview of whether findings across reviews are consistent, contradictory or context-dependent.^49 50^ Given the growing number of reviews on the involvement of older adults in research, an umbrella review offers a rigorous method to map, assess and synthesise this body of evidence.

This umbrella review constitutes the first phase of a larger mixed-methods project with the overarching aim of identifying and building consensus on strategies to strengthen the involvement of older adults as active partners in healthcare research. A separate protocol outlining the overall project design has been developed.^51^

To determine whether an umbrella review on this topic already existed, a preliminary search was conducted and no umbrella review was identified that synthesised review-level evidence on the involvement of older adults as research partners or co-creators across participatory approaches. Although scoping and systematic reviews on public involvement in healthcare research in general exist,^52 53^ these do not offer a comprehensive synthesis focused on older adults.

This review will be conducted in accordance with the Joanna Briggs Institute (JBI) methodology for umbrella reviews^48^ and reported in accordance with the Preferred Reporting Items for Overviews of Reviews guidelines.^54^ The protocol itself has been developed in line with the Preferred Reporting Items for Systematic Review and Meta-Analysis Protocols statement.^55^ It is registered in PROSPERO (registration number: CRD420251064947). The review process, including the systematic search and data analysis, is scheduled for January 2026 to March 2026. Any amendments to the protocol will be documented and described in the final report.

### Eligibility criteria

The review questions and eligibility criteria were guided by the PEO (Population, Exposure, Outcome) framework.^56^ This framework was used to define the key elements of the review scope: older adults (Population), their involvement as active partners in research (Exposure), and the reported forms, models, terminology, benefits and challenges of such involvement (Outcome) (table 1). ‘Older adults’ is defined as individuals aged 60 years or older, in accordance with the WHO’s global classification.^17^ This age threshold ensures international comparability and reflects the diversity of ageing experiences. Reviews conducted in any health care, social care or community-based research setting will be included.

**Table 1:** PEO framework used to structure eligibility criteria

Component	Description
P (Population)	Older adults (generally defined as aged 60 years or older).
E (Exposure)	Involvement in research as active partners, co-creators or co-researchers.
O (Outcome)	Reported roles, experiences, terminology, frameworks, benefits or challenges of involvement.

### Data sources and search strategy

A comprehensive three-phase search strategy will be employed to identify relevant literature syntheses for inclusion in this umbrella review, in accordance with the JBI methodology for umbrella reviews.^48^ The search will be designed to capture both the type of review (eg, systematic reviews, meta-analyses, scoping reviews) and the subject matter—namely, the involvement of older adults as co-researchers, co-creators, or active participants in research.

Initially, searches were conducted in Epistemonikos, a multilingual database of health evidence; PROSPERO, an international systematic review registry; and the Cochrane Library, to inform the development of the search strategy and to search for published or ongoing reviews^48 49^ (see search report in online supplemental material 1). No current or underway umbrella reviews on the topic were identified. Following this, broad exploratory searches were run in bibliographic databases (eg, MEDLINE, CINAHL) to understand how concepts related to older adults and their involvement in research were described in the literature. These searches used combinations of terminology for different review types and terms related to older adults and co-research activities to assess how review-level evidence was represented. Titles, abstracts and indexing terms from retrieved records were analysed to identify additional terminology. In the second phase, combinations of controlled vocabulary (eg, MeSH terms) and free-text keywords will be used to develop a strategy broad enough to capture all relevant evidence relating to the three main concepts: (1) review types (eg, ‘systematic review’, ‘meta-analysis’, ‘scoping review’, ‘umbrella review’); (2) the population of interest (eg, ‘older adults’, ‘elderly’, ‘aging’) and (3) participatory approaches (eg, ‘co-creation’, ‘co-production’, ‘co-research’, ‘patient and public involvement’, ‘citizen science’, ‘community-based participatory research’).

Predefined search filters for review types will be utilised when applicable. For databases with no defined filters, the terms for review types will be directed towards title fields, since most authors use these terms in titles to identify the article as a type of evidence synthesis. A final search strategy will be tested on MEDLINE and CINAHL. Search strategies will thereafter be adapted to the indexing structure and syntax of each database and platform. The following six bibliographic databases will be searched: APA PsycInfo (ProQuest), CINAHL with FullText (EBSCOhost), MEDLINE ALL (Ovid), Scopus, Sociological Abstracts (ProQuest) and Web of Science Core Collection. Full search strategies for all databases will be reported, to ensure transparency and reproducibility.

In the third and final phase, the reference lists of included reviews will be manually screened (citation chasing) to identify any additional relevant publications that may not have been captured by the database searches.

A preliminary MEDLINE strategy is presented in table 2. A test run on 28 November 2025 yielded 1816 records (see full search results in online supplemental material 3). A pilot screening of a sample of these records confirmed that the strategy successfully retrieved studies relevant to the review aim, indicating that the search terms and structure were appropriate for identifying eligible systematic reviews.

**Table 2:** Preliminary search strategy for Medline

	Query
S1	exp Citizen Science/ or exp Community-Based Participatory Research/
S2	(“co-author*” or “co-creat*” or “co-design” or “co-produc*” or “co-research*” or “citizen science*” or “inclusive research” or “patient involv*” or “public involv*” or “patient particip*” or “user involv*").tw,kf.
S3	((involv* or engag* or collaborat* or partner* or particip*) adj5 research).tw,kf.
S4	S1 OR S2 OR S3
S5	exp “Aged, 80 and over”/ or Middle Aged/ or Aged/
S6	(ageing or aging or centarian* or centenarian* or elder* or eldest or nonagenarian* or octagenarian* or octogenarian* or “older adult*” or “old age*” or “older man” or “older men” or “oldest old” or “older people” or “older patient*” or “older person*” or “older woman” or “older women” or senior* or senium or septuagenarian* or sexagenarian* or septuagenarian* or supercentenarian* or “very old”).tw,kf.
S7	S5 OR S6
S8	“Systematic Review”/ or “Scoping Review”/ or Review/
S9	(“meta-analysis” or metaanalysis or “meta-ethnography” or “metaethnography” or “meta-synthesis” or metasynthesis or “narrative synthesis” or overview or “research evidence” or review*).ti.
S10	S8 OR S9
S11	S4 AND S7 AND S10

### Study selection

All search results will be directly imported into Covidence software (https://www.covidence.org/), a web-based tool for systematic review management, which will be used to manage records and facilitate the blinded screening process. Duplicate records will be identified and removed. Two reviewers will screen titles and abstracts independently, to minimise bias. Before complete screening, a pilot test of the inclusion and exclusion criteria will be conducted on a subset of records, to ensure consistency in their application. Articles deemed potentially eligible will undergo full-text review, also conducted independently by the same two reviewers.^48^ Any disagreements at any stage will be resolved through discussion, or by involving a third reviewer if consensus cannot be reached. Reasons for exclusion at the full-text stage will be documented. To avoid potential conflicts of interest, reviewers will not screen any reviews in which they are listed as authors or have otherwise been involved. In such cases, an independent team member with no prior involvement in the review will make the inclusion decision. The final selection of included reviews will be discussed and agreed on collectively by the research team, to ensure transparency and consistency.

### Critical appraisal

The methodological quality of the included systematic reviews will be assessed using the JBI Critical Appraisal Checklist for Systematic Reviews and Research Syntheses (table 3).^48^ The JBI tool was selected because it is specifically designed for umbrella reviews and is suitable for appraising diverse types of review methodologies, including qualitative, quantitative and mixed-methods reviews. Given the anticipated heterogeneity of review designs in this field, JBI was considered more appropriate than tools primarily developed for intervention-focused systematic reviews such as A MeaSurement Tool to Assess Systematic Reviews 2 (AMSTAR-2). The Grading of Recommendations Assessment, Development and Evaluation (GRADE) will not be applied, as the aim is to synthesise conceptualisations, roles and reported experiences rather than pooled intervention effects. However, while the JBI checklist will guide the formal methodological appraisal, additional review-level methodological characteristics—such as search comprehensiveness, transparency of eligibility criteria and clarity of synthesis methods—will also be systematically extracted and considered to provide a more nuanced assessment of review quality.

**Table 3:** JBI critical appraisal checklist for systematic reviews and research syntheses^48^

Item	Yes	No	Unclear	Not applicable
Is the review question clearly and explicitly stated?	☐	☐	☐	☐
Were the inclusion criteria appropriate for the review question?	☐	☐	☐	☐
Was the search strategy appropriate?	☐	☐	☐	☐
Were the sources and resources used to search for studies adequate?	☐	☐	☐	☐
Were the criteria for appraising studies appropriate?	☐	☐	☐	☐
Was critical appraisal conducted by two or more reviewers independently?	☐	☐	☐	☐
Were there methods to minimise errors in data extraction?	☐	☐	☐	☐
Were the methods used to combine studies appropriate?	☐	☐	☐	☐
Was the likelihood of publication bias assessed?	☐	☐	☐	☐
Were recommendations for policy and/or practice supported by the reported data?	☐	☐	☐	☐
Were the specific directives for new research appropriate?	☐	☐	☐	☐

JBI, Joanna Briggs Institute.

Two reviewers will independently conduct the appraisal. Disagreements will be resolved through discussion, with a third reviewer consulted if needed. Reviewers will not appraise reviews in which they are authors; in such cases, an independent team member will undertake the appraisal.

Predefined thresholds will be applied to categorise methodological quality (0%–49% low, 50%–74% moderate, ≥75% high). The results of the critical appraisal will be presented visually using a traffic light scheme, with each item coded green for ‘yes’, red for ‘no’, yellow for ‘unclear’, or blank for ‘not applicable’. A descriptive summary of the methodological quality across the included reviews will be provided in the final report.

### Data extraction

Data will be extracted independently by two reviewers using a standardised data extraction form developed for this umbrella review (see online supplemental material 2). The form is based on the JBI guidance for umbrella reviews and includes key domains such as review characteristics, population details, description of participatory approaches, methodological features, outcomes and findings. The form will be piloted on a purposive sample of three reviews, to ensure clarity, relevance and consistency in the data extraction process.

Data will be extracted based on the type of study—quantitative, qualitative or mixed-methods. For reviews without meta-analyses, we will extract the number of included studies, total sample size, reported findings and narrative conclusions. For reviews with meta-analyses, we will extract the number of included studies, total and study-specific sample sizes and events, effect estimates with 95% CIs, and any reported quality assessments or GRADE evaluations (if available).^49^

Data extraction will include descriptions of involvement approaches, terminology, frameworks and reported benefits and challenges. When reviews also report older adults’ own reflections or experiences of acting as research partners, these will be extracted and synthesised. Incorporating experiential accounts provides additional insight into the conditions under which involvement is perceived as meaningful or insufficiently realised. If few or no reviews report such experiences, this will be noted as a gap in the existing evidence base.

### Data summary

Extracted data will be synthesised using a narrative and thematic approach.^48^ Qualitative data analysis software such as NVivo or equivalent will be utilised. The thematic synthesis will be explicitly guided by the review’s research questions and will focus on: (1) how older adults have been involved as active partners in research, (2) the terminology and conceptual frameworks used to describe such involvement and (3) the reported benefits and challenges associated with this involvement. Study characteristics will be summarised in a tabular format, providing an overview of the evidence base.

Where meta-analyses are included, we will present the summary estimates as reported. In the absence of meta-analyses, key findings from narrative syntheses will be thematically organised to construct an overarching synthesis of the literature. A focused summary of the conceptual models and participatory approaches identified across reviews will be developed to map the landscape of involvement strategies in research with older adults.

### Patient and public involvement

Older adults have been involved as public contributors from the early stages of this planned umbrella review. Their lived experience and insights have informed the development of the research questions, the selection of relevant terminology and the framing of the review objectives. They have reviewed the protocol for clarity and relevance, ensuring that the study design reflects issues of importance to older adults. Their involvement goes beyond consultation. A key aim of the project is to strengthen the role of older adults as co-creators, co-researchers and co-authors. By supporting their active participation throughout the review process—from data interpretation to co-development of dissemination materials—we seek to promote equitable and meaningful collaboration that values experiential knowledge on par with academic expertise.

This involvement is grounded in the principles of co-creation, mutual learning and empowerment, with a strong focus on promoting relevance, transparency and accessibility in the research process. Public contributors are included as co-authors of the protocol and are affiliated as independent public contributors from Sweden.

## Ethics and dissemination

As this umbrella review is based solely on previously published literature, no ethical approval is required. The review constitutes a foundational phase of a mixed-methods research project. Findings from the umbrella review will inform the design of the subsequent studies by helping to refine terminology, identify key domains for consensus and ensure that the involvement of older adults is meaningfully integrated.

The findings will be disseminated through a peer-reviewed open access publication and presentations at academic and relevant conferences. Older public contributors have played an active role in shaping this protocol and will remain engaged throughout the review and subsequent phases. Their continued involvement will support the development of dissemination materials that are accessible and relevant to both academic and non-academic audiences.

## Supplementary material

10.1136/bmjopen-2025-114885 online supplemental file 1

10.1136/bmjopen-2025-114885 online supplemental file 2

10.1136/bmjopen-2025-114885 online supplemental file 3
