# Supplementary material for: Older adults as active research partners: protocol for an umbrella review
Source: BMJ Open. 2026 Mar 18;16(3):e114885. doi: 10.1136/bmjopen-2025-114885 (PMC13007083; doi:10.1136/bmjopen-2025-114885)
Supplement: online supplemental file 1 [file bmjopen-16-3-s001.pdf]

Supplemental material 1 - Epistemonikos exploratory search report

Database: Epistemonikos
Platform: https://www.epistemonikos.org
Search date: 14 November 2025
Search type: Exploratory mapping search (Phase 1)

Search description

The search was performed in two steps. In the first step, a broad exploratory search was conducted using general terms related to older adults and participatory research to identify how these concepts were described in the literature. In the second step, the search was refined by adding review-type terms to focus on review-level evidence and assess the coverage of relevant syntheses.

Search strings

Step 1 – Broad exploratory search:

("older adults" OR "older people" OR "older persons" OR elderly OR ageing OR aging OR geriatric)

AND

("participatory research" OR "patient involvement" OR "public involvement" OR "co-production" OR "co-creation" OR "research partner" OR "co-research" OR "patient engagement")

Step 2 – Exploratory search including review-type terms:

("older adults" OR "older people" OR "older persons" OR elderly OR ageing OR aging OR geriatric)

AND

("participatory research" OR "patient involvement" OR "public involvement" OR "co-production" OR "co-creation" OR "research partner" OR "co-research" OR "patient engagement")

AND

("systematic review" OR "literature review" OR "scoping review" OR "rapid review" OR "integrative review" OR "review of literature" OR "narrative synthesis" OR "narrative review" OR "umbrella review" OR "meta-ethnography" OR "meta analysis" OR "meta-analysis" OR "meta synthesis" OR "meta-synthesis")

Search results summary

Step	Description	Records retrieved
1	Initial broad search (no filters)	2,903
2	Broad search – 2015–2025	2,353
3	With review-type terms (no filters)	455
4	With review-type terms – 2015–2025	427
