# Supplementary material for: Older adults as active research partners: protocol for an umbrella review
Source: BMJ Open. 2026 Mar 18;16(3):e114885. doi: 10.1136/bmjopen-2025-114885 (PMC13007083; doi:10.1136/bmjopen-2025-114885)
Supplement: online supplemental file 2 [file bmjopen-16-3-s002.pdf]

Supplemental material 2 - Data Extraction Table

Box 1. Data Extraction Table.

**Item / Field**	**Explanation / Instructions**
Citation (Author, Year)	Full citation for the review, including author(s) and year of publication.
Aims / Objectives	The main aim(s) or objective(s) of the review as stated by the authors.
Review type / Design	Type of review conducted (e.g. systematic review, scoping review, meta-analysis).
Population / Context	Characteristics of the population studied and the context (e.g. older adults in community care).
Phenomenon of Interest	Focus of the review – what was being studied or explored (e.g. co-production, PPI).
Databases / Sources Searched	Databases and sources used in the review (e.g. PubMed, CINAHL, grey literature).
Year Range of Included Studies	Time period covered by the included studies (e.g. 2005–2023).
Number of Included Reviews / Studies	Total number of studies or reviews included in the umbrella review.
Study Types Included	Types of studies included (e.g. qualitative, quantitative, mixed-methods).
Country / Region(s) Covered	Geographic location(s) of the studies reviewed.
Quality Appraisal Method	Tool or framework used to assess quality (e.g. AMSTAR, CASP).
Quality Rating / Appraisal Result	Summary of the quality appraisal results or ratings.
Method of Synthesis / Analysis	Approach used to synthesize data (e.g. thematic synthesis, meta-aggregation).
Key Findings / Themes	Summary of main findings or emergent themes.
Key Recommendations / Implications	Recommendations for practice, policy, or research based on findings.
Terminology / Concepts Used	Specific terms or concepts that were central to the review.
Theoretical Frameworks / Models	Any theoretical frameworks or models used in the review.
Notes / Comments	Additional notes or comments relevant for interpretation or inclusion.
