# Supplementary material for: Older adults as active research partners: protocol for an umbrella review
Source: BMJ Open. 2026 Mar 18;16(3):e114885. doi: 10.1136/bmjopen-2025-114885 (PMC13007083; doi:10.1136/bmjopen-2025-114885)
Supplement: online supplemental file 3 [file bmjopen-16-3-s003.pdf]

Supplemental material 3 – Medline search report

MEDLINE (Ovid) n=1,816
Date of search: November 28, 2025
Search Mode: Advanced Search

	**Query**	**Results**
S1	exp Citizen Science/ or exp Community-Based Participatory Research/	7,356
S2	("co-author*" or "co-creat*" or "co-design" or "co-produc*" or "co-research*" or "citizen science*" or "inclusive research" or "patient involv*" or "public involv*" or "patient particip*" or "user involv*").tw,kf.	37,485
S3	((involv* or engag* or collaborat* or partner* or particip*) adj5 research).tw,kf.	104,975
S4	S1 OR S2 OR S3	139,091
S5	exp "Aged, 80 and over"/ or Middle Aged/ or Aged/	6,052,189
S6	(ageing or aging or centarian* or centenarian* or elder* or eldest or nonagenarian* or octagenarian* or octogenarian* or "older adult*" or "old age*" or "older man" or "older men" or "oldest old" or "older people" or "older patient*" or "older person*" or "older woman" or "older women" or senior* or senium or septuagenarian* or sexagenarian* or septuagenarian* or supercentenarian* or "very old").tw,kf.	939,330
S7	S5 OR S6	6,467,584
S8	"Systematic Review"/ or "Scoping Review"/ or Review/	3,758,962
S9	("meta-analysis" or metaanalysis or "meta-ethnography" or "metaethnography" or "meta-synthesis" or metasynthesis or "narrative synthesis" or overview or "research evidence" or review*).ti.	1,061,576
S10	S8 OR S9	4,084,009
S11	S4 AND S7 AND S10	1,816
