# Supplementary material for: Older adults as active research partners: protocol for an umbrella review
Source: BMJ Open. 2026 Mar 18;16(3):e114885. doi: 10.1136/bmjopen-2025-114885 (PMC13007083; doi:10.1136/bmjopen-2025-114885)
Supplement: Supplementary file 4 [file bmjopen-2025-114885.reviewer_comments.pdf]

PEER REVIEW HISTORY

BMJ Open publishes all reviews undertaken for accepted manuscripts. Reviewers are asked to complete a checklist review form and are provided with free text boxes to elaborate on their assessment. These free text comments are reproduced below.

ARTICLE DETAILS

Title (Provisional)

Older Adults as Active Research Partners: Protocol for an Umbrella Review

Authors

Hedqvist, Ann-Therese; Strandberg, Susanna; Holmberg, Bodil; Niklasson, Joakim; Violasdotter Nilsson, Paola; Carlsson, Willemo; Andreassen, Maria; Bergstrand, Sara; Holmberg, Mats; Nilsen, Charlotta

---

VERSION 1 - REVIEW

Reviewer **1**

Name **Satoh, Michihiro**

Affiliation **Tohoku Medical and Pharmaceutical University, Division of Public Health, Hygiene and Epidemiology, Faculty of Medicine**

Date **04-Feb-2026**

COI **None**

Thank you for the opportunity to review this protocol for an umbrella review on older adults' involvement as active partners in research. The topic may be important, given the growing emphasis on participatory research approaches and the need to understand how older populations are engaged in research processes. However, I have several concerns that should be addressed to strengthen the manuscript.

1. Another umbrella review used AMSTAR-2 and the Grading of Recommendations, Assessment, Development and Evaluation (GRADE) criteria to assess methodological quality. The authors should provide a rationale for why the JBI Critical Appraisal Checklist will be used instead.

2. The review is scheduled for November 2025 to December 2025. However, if the authors plan to start the review after publishing this protocol paper, the timeframe should be extended.

3. The number of outcomes may be excessive.

**Reviewer**	**2**
**Name**	**Yong, Kenneth**
**Affiliation**	**Prince of Wales Hospital, Department of Nephrology**
**Date**	**28-Feb-2026**
**COI**	**None**

Dear BMJ Open Editor,

Thank you for the opportunity to review again the manuscript titled "Older Adults as Active Research Partners: Protocol for an Umbrella Review; manuscript ID bmjopen-2025-114886) This is a protocol for a proposed umbrella review of current literature of engagement of older adults (>60yo) as active partners (creators, participants or designer) in research. The described methodology is sound and well:

1) It is not clear from the introductory summary about the reasons why there appear to be barriers towards older adult engagement in research and hence the rationale of this review. It would be helpful to perhaps include some discussion about factors such as physical/cognitive health, logistical hurdles, institutional factors as well as structural and/or ageist barriers. Otherwise it appears that there is an assumption that the reader has a significant amount of knowledge in this subject matter already.

2) Only one older adult was actively involved as a co-creator: is this sufficient? Would it be not more helpful to have at least two or three such older adults involved in the design of this study given the great degrees of heterogeneity and specific need across this population.

3) It seems like there is no mention about the proposed outcomes that this review will report ie. quantitative or qualitative results which would tie in with the factors I mentioned in my 1st comment? Ie. there is a research question posed at trying to discern above barriers and therefore find ways to overcome them? As a reader this would make this manuscript much more interesting and relevant towards the topic in question.

VERSION 1 - AUTHOR RESPONSE

Response to Reviewer 1

We sincerely thank the reviewer for the careful reading of our manuscript and for the constructive comments. We have addressed each point in detail below.

Comment 1

Another umbrella review used AMSTAR-2 and the GRADE criteria to assess methodological quality. The authors should provide a rationale for why the JBI Critical Appraisal Checklist

will be used instead.

Response

We appreciate this important methodological comment. We have now clarified and expanded the rationale for using the JBI Critical Appraisal Checklist for Systematic Reviews and Research Syntheses.

The JBI checklist was selected because it is specifically developed for umbrella reviews and accommodates both quantitative and qualitative systematic reviews, which is particularly relevant given the expected heterogeneity of review types in this field. Unlike AMSTAR-2, which is primarily tailored to intervention reviews, the JBI tool allows appraisal of mixed-methods and narrative syntheses. We have added a paragraph in the Methods section to clarify this rationale.

Comment 2

The review is scheduled for November 2025 to December 2025. However, if the authors plan to start the review after publishing this protocol paper, the timeframe should be extended.

Response

We thank the reviewer for highlighting this. We have revised the timeline to reflect a realistic timeframe for completion of the search, screening, appraisal, and synthesis stages. The timeframe has now been extended.

Comment 3

The number of outcomes may be excessive.

Response

Thank you for this observation. We have revised the manuscript to remove the term “outcomes” and clarified that this umbrella review does not assess predefined clinical or quantitative outcomes. Instead, the synthesis focuses on how older adults are involved as active research partners, the terminology and conceptual frameworks used to describe such involvement, and the reported benefits and challenges associated with it. This revision aligns the data synthesis more clearly with the review’s research questions.

Response to Reviewer 2

We thank the reviewer for the thoughtful and constructive feedback.

Comment 1

It is not clear why there appear to be barriers towards older adult engagement in research and hence the rationale of this review.

Response

We agree that the introduction required clearer articulation of known and potential barriers to older adults' engagement. We have now expanded the background section to explicitly describe structural, institutional, logistical, and health-related barriers, including ageism, digital exclusion, cognitive and physical limitations, and power imbalances in research settings.

Comment 2

Only one older adult was actively involved as a co-creator: is this sufficient?

Response

We appreciate this important comment. While one older adult has been actively involved as a co-creator in developing the protocol, this umbrella review synthesises existing review-level evidence and does not involve primary data collection. Nevertheless, we acknowledge the importance of broader representation and have clarified that additional older contributors will be invited to engage during later phases of the broader mixed-methods project.

Comment 3

There is no mention about the proposed outcomes that this review will report.

Response

We appreciate this comment and have clarified the scope of the review. As the aim of this umbrella review is to synthesise conceptualisations, roles, and reported experiences of involving older adults as research partners, we do not evaluate predefined intervention outcomes. The Data Summary section has been revised to explicitly state that the thematic

synthesis will be guided by the research questions and will focus on involvement processes, terminology, frameworks, and reported benefits and challenges. This clarification has been incorporated in the revised manuscript (Data Summary section).
